# Availability of ‘Do-It-Yourself’ orthodontics in the United Kingdom

**DOI:** 10.1177/14653125211021607

**Published:** 2021-06-06

**Authors:** Annabelle Carter, Susan Stokes

**Affiliations:** Leeds Dental Institute, Leeds, UK

**Keywords:** orthodontics, Do-It-Yourself braces, teledentistry

## Abstract

**Objective:**

To identify the number of companies providing Do-It-Yourself (DIY) orthodontics and explore information available on websites for DIY brace providers operating in the UK.

**Design:**

Web search and review of websites providing DIY braces.

**Setting:**

Leeds, UK.

**Methods:**

A Web search was completed in November 2020 and April 2021 of all companies providing DIY braces for UK consumers. Each website was evaluated, and the following data collected: name; year started operating; costs; process; involvement of a dental professional; average ‘treatment’ length; retention; consent process; information on risks and benefits; aligner material; social media presence; age suitability; and consumer ratings on Trustpilot. Quality of website information was assessed via the DISCERN tool.

**Results:**

Seven DIY orthodontic companies were operating in the UK. Websites reviewed revealed the following: product costs were in the range of £799–£1599, ‘treatment’ length quotes were in the range of 4–12 months; Trustpilot reviews were in the range of 1.6–4.8 stars; and websites claimed their aligners were suitable for individuals with an age range of 12–18 years. Quality of content regarding risks described on websites varied, and there was limited information regarding involvement of a dental professional. Quality of websites information scored ‘poor’ or ‘very poor’ on the DISCERN scoring.

**Conclusions:**

There has been an increase in the number of DIY orthodontic companies operating in the UK over the last three years. There is a need to determine whether these products constitute dental treatment in their own right. If so, it is crucial to ensure these are regulated appropriately with adequate information available to satisfy informed consent and have greater transparency over dental professional involvement to safeguard the public.

## Introduction

Do-It-Yourself (DIY) orthodontics is a newly emerging phenomenon and is a process whereby a person can gain access to orthodontic treatment and can have clear aligners sent to their home, so they can align their teeth without seeing or being supervised by an orthodontist or dentist. It can also be called direct-to-consumer (DTC) orthodontics (Kravitz et al., 2016; Wexler et al., 2020). The term ‘consumers’ will be used for people who use these websites and products instead of ‘patients’.

There has been a recent surge in the popularity of DIY orthodontic products. The COVID-19 pandemic has led to limited face-to-face appointment availability and increasing waiting times. Furthermore, people may be reluctant to attend clinical settings. Meanwhile, social media continues to drive an increasing aesthetically centric society. One of the companies which provides DIY braces has stated that enquiries about their products have tripled since the first COVID-19 UK March lockdown (This is Money, 2020).

Other appeals of DIY treatment include that it is a fraction of the cost of conventional practice-based orthodontics, frequently quoted to be 60%–70% cheaper, as well as the convenience of not having to attend and travel for appointments (Bous et al., 2021, Kravitz et al., 2016; O’Dowd, 2020; Okuda et al., 2021, Wexler et al., 2020). However, concerns have been raised by multiple international specialist dental societies about issues surrounding assessment of suitability, consent, safety, regulation and scope of practice (American Association of Orthodontists, 2019; American Dental Association, 2020; British Orthodontic Society, 2020a, 2020b; O’Dowd, 2020).

Marketing, advertising, information sharing and consent procedures of DIY aligners is primarily done online. Product websites are where consumers go to seek further information to make the decision to purchase, particularly during the COVID-19 pandemic, as the already few DIY aligner face-to-face shops closed. This means information available on these websites should be high quality for consumers to make well-informed choices surrounding their oral health. However, these websites undergo little regulation and quality is rarely reviewed.

A literature review revealed limited literature regarding the information available and quality of such information on DIY-orthodontic company websites, with only two papers reviewing and discussing this (Meade and Dryer, 2020, 2021). This is of interest, as the information provided on these websites is what members of the public use to decide whether to undertake DIY ‘treatment’. Tools previously used in the literature to review the quality of websites include LIDA, JAMA and Discern (Meade and Dryer, 2020, 2021; Parekh and Gill, 2014).

The primary aim of the study was to identify the number and availability of DIY-orthodontic companies operating in the UK (via an Internet search). The secondary aim was to review information on the company websites, including: year company started operating in the UK; treatment process; remote monitoring availability; dental professional involvement; if noted General Dental Council (GDC) number visible; treatment length; treatment regimens (i.e. day and/or night aligners available); fitness pre-assessment; requirement for pre-treatment dental health; age suitability stated; discussion of risks/harms; consent details; aligner materials; retainer protocols; need for life-long retention; pricing; social media presence; consumer ratings on Trustpilot; and the quality of website information via a quality assessment tool.

## Materials and methods

An Internet search was completed in Leeds, UK in November 2020 and April 2021. The following keywords were used in Internet search engines: ‘Do-It-Yourself braces/aligners’, ‘clear braces at home’, ‘home orthodontics’, ‘postal braces’, ‘direct to consumer braces/orthodontics’, ‘tele-dentistry/orthodontics’.

The following inclusion criteria were used to identify websites: websites for DIY braces and operating in the UK. Companies operating outside the UK or not in the English language were excluded.

The information collected was readily available on the websites of companies providing DIY-orthodontic products. It was beyond the scope of the purpose of this project to obtain more information by directly contacting the company sales team. It is likely that there is further information disclosed to the consumer should they wish to purchase DIY braces.

The quality of website information was reviewed by the DISCERN tool, a validated quality of information instrument used commonly in studies (Charnock et al., 1999). It consists of 16 questions, each response having a Likert-scale-type score of 1–5. The final possible highest score is 80; the lower the score, the poorer the quality of health information for patients (DISCERN, 1997).

### Data collection

For all company websites for DIY braces, the following data were collected and collated onto a Microsoft Excel spread sheet: name; year company started operating in UK; costs; dental professional involvement; GDC number visible; treatment process; remote monitoring discussed; treatment lengths; retention mentioned; retainers included or additional price; need for pre-treatment dental health mentioned; fitness pre-assessment; consent process; description of risks and benefits; materials of aligners; social media presence; age suitability; consumer ratings on Trustpilot; and DISCERN scoring.

## Results

*Companies*

The web-search using the popular search engine Google revealed that there were seven companies offering DIY braces to people in the UK. These companies included Smile Direct Club, Wonder Smile and Smile Life. There were 11 companies in total, but four were only available in the USA.

*Operations in the UK*

Information about operations in the UK is shown in Table 1.

**Table 1.** Summary of website information regarding cost, treatment length, wear regimens, retention and aligner material.

Company website number	Year operations started in UK	Cost	Treatment length and wear regimens	Retention	Aligner material
1	2020	£1400 with free whitening kit	6 months for 22 hour wear	Additional £200	No information
2	2019	£1539	4–6 months for 22 hour wear 10 months for night-time (10 hour wear)	Additional £80	BPA-free resin
3	2019	£1567	6 months for 22 hour wear	No information	No information
4	2019	£1399	6 months for 22 hour wear 9 months for night-time 10 hour wear	Additional £60	BPA-free and phthalate-free resin
5	2019	£1390	4–10 months for 22 hour wear	Additional £99	BPA-free resin
6	2018	£799–£1149	6–11 months for 22 hour wear 8–12 months for night-time 8–10 hour wear	Included	BPA-free resin
7	2017	£1350	5–9 months for 22 hour wear	Included	BPA-free resin

*Cost*

The pricing of braces quoted on websites was in the range of £799–£1599 for a one-off payment. All companies offered a monthly finance option. All companies claimed to be either 60% or 70% cheaper than conventional orthodontist-led braces. All make claims of ‘traditional’ orthodontic costs, in the range of £4200–£5000, as a comparison. More information is in Table 1.

*Process*

The ‘treatment process’ was described for three of the companies (companies 2, 4 and 6) as the following: impressions taken by customer at home; impressions reviewed by dentist/orthodontist; and sequence of clear aligners posted to the customer’s home. Four companies (companies 2, 4, 6 and 7) offered the option to attend their shops to have an intraoral 3D scan instead of home impressions. Company 1 described at home impressions, before and after 3D visuals, aligners posted and remote monitoring via a scan box linked to an app with dental support.

Company 3 described at home impression taking by the consumer and aligners posted but did not mention whether impressions were checked by a dental professional.

For company 7, the customer submitted photos of their smile via a smartphone app. These were reviewed by a dentist, followed by an e-consultation (it did not specify with whom), then customers could take at-home impressions or have an intraoral scan at their London base, and have aligners posted.

Company 5’s website stated a dentist would take a 3D intraoral scan, give advice, plan the aligner treatment and aligners would be sent to the customer’s home.

*Monitoring*

Six company websites (companies 1, 2, 4–7) mentioned remote monitoring of aligners as part of their process, either via ‘virtual check-ins’ or a ‘movement tracker app’ with photos being submitted every two weeks. One company website (company 3) had no mention of monitoring.

*Treatment length and wear regimens*

The average ‘treatment’ lengths were in the range of 4–11 months. Three companies offered night-time only aligner options. More information on treatment length and wear regimens is in Table 1.

*Retainers*

The need for retention and retainers for successful and long-lasting results to prevent relapse was mentioned on six of the seven websites (the authors could find no information on company 3’s website). More information on retention is in Table 1.

*Dental professionals*

Information on whether a dentist or orthodontist was involved was often vague, unclear at which stage it would be or how much involvement they had. Four company websites said they had UK-registered dentists on their team; one said they used dentists and orthodontists based in Europe; one said that it was orthodontist supervised but gave no further information about the orthodontists used. Another company mentioned a ‘dental team’ but no further clarification. There were only two websites (companies 1 and 7) that contained named dentists, with company 7 having GDC registration numbers available on the website.

*Consent and risks noted*

There was limited information on the consent process involved on the websites reviewed. None contained any detail of risks and adverse outcomes. Consent may or may not take place before purchasing the aligners but we cannot comment on the quality or robustness of this process.

*Pre-assessment (i.e. need for dental health)*

Three websites (companies 1, 5 and 7) advise the need to be ‘dentally fit’ and advise to see a dentist for check-ups. No other websites noted the need for pre-treatment dental health or a dental check-up. The company 7 website also discussed sharing recent X-rays and Basic Periodontal Examination scores with them. However, sharing of this information is still at the consumer’s discretion, so it is unclear whether they can truly safeguard and check that a customer is safe when there is no face-to-face dental assessment before aligner provision.

*Quality of website information, DISCERN score*

All websites scored poorly or very poorly according to the DISCERN score and the predetermined scales where 16–26 was very poor, 27–38 was poor, 39-50 was fair, 51–62 was good and > 63 was excellent (Charnock, 1999; Meade and Dreyer, 2021).

Information on aligner material, Trustpilot ratings, social media presence, age suitability and DISCERN score is listed in Table 2.

**Table 2.** Information summary of each company regarding reviews, social media presence, age suitability and DISCERN score for quality of website information.

Company website number	Trustpilot reviews (stars out of 5)	Social media presence	Age suitability (years)	Quality of website information: DISCERN score
1	4.9 (320 reviews)	YouTube, Facebook, Instagram, Twitter	18+	25, i.e. very poor
2	4.3 (3048 reviews)	YouTube, Facebook, Instagram, Twitter	12+	28, i.e. poor
3	4.6 (116 reviews)	YouTube, Facebook, Instagram, Twitter	18+	27, i.e. very poor
4	3.2 (1 review)	Facebook, Instagram	12+	28, i.e. poor
5	4.4 (203 reviews)	Facebook, Instagram	18+	38, i.e. poor
6	4.7 (153 reviews)	YouTube, Facebook, Instagram	14+	28, i.e. poor
7	4.8 (390 reviews)	YouTube, Facebook, Instagram, Twitter	18+	34, i.e. poor

## Discussion

All seven companies have appeared within the last three years, which highlights the novel and fast-growing DIY orthodontics market.

With patients increasingly looking to achieve a perfect smile at a competitive price, the number of companies offering DIY braces is anticipated to rise. The COVID-19 pandemic has impacted on access to orthodontic appointments (fallow time, social distancing, and concern around travel and attendance at clinics), meaning increasing numbers of consumers are tempted to undertake DIY-orthodontics compared to conventional orthodontist or dentist-led treatment. A recent study of 1141 questionnaire participants found that convenience and cost were the main reasons why people chose DIY orthodontics, with 26.6% participants more likely to pursue DIY-orthodontics due to the COVID-19 pandemic (Bous et al., 2021). Cost and convenience being the main reasons for choosing DIY orthodontics was supported by another research survey by Okuda et al. (2021). This questionnaire also found that as household income, age and education increased, people were more likely to choose DIY aligners, which is different from common dentist perceptions and should help societies target public education (Okuda et al., 2021). A British Orthodontic Society (BOS) survey found 65% of orthodontists were concerned increasing numbers of patients would seek DIY braces both during and after lockdown (BOS, 2020b).

The variability of risk-related information on company websites found in this project reinforces concerns voiced by specialist dental societies. As well as variations in the ‘treatment’ process, including lack of face-to-face assessment by a suitably qualified dental professional and uncertain levels of clinician oversight, which could mean dental disease is missed, or such products may be inappropriate or unsafe to use and have negative consequences on oral health (BOS, 2020a; O’Dowd, 2020). The BOS, alongside the Oral Health Foundation, launched a campaign called ‘Safe Brace’ with the aim of reaching prospective users of DIY brace companies and ensuring awareness of the risks to dental health before embarking on such treatments (BOS, 2020a, 2020b, 2020c; O’Dowd, 2020). Concerns have been echoed internationally, by the American Dental Association and the American Association of Orthodontists, who also have concerns regarding the ‘potential harm to patients’ and risk of ‘potentially irreversible and expensive damage such as tooth and gum loss, changed bites, and other issues’ (American Association of Orthodontists, 2019; American Dental Association, 2020; Wexler et al., 2020).

Without regulation, consumers are at risk of unmet expectations, complications putting their occlusion and oral health at risk, and other undesired effects. These can prove difficult, time-consuming and costly to amend through corrective treatment. A study by Wexler et al. (2020) investigated customers’ experiences with DIY aligners via a survey. It found that 87.5% of respondents were satisfied with their DIY aligners, but 6.6% needed to visit their dentist due to significant complications (Wexler et al., 2020).

The GDC, the UK regulator of dentistry, has a statutory objective to protect the public and maintain public confidence in dental services. They state any form of dental treatment in the UK needs to be undertaken by and taken responsibility for by a GDC-registered dentist. These companies may be in breach of the Dentists Act 1984, if it is determined these aligners count as the practice of dentistry, as only two websites contained information of GDC-registered dentists. Of note, one website (company 1) has a dentist affiliated with them as the face of the website and describes itself as a ‘hybrid business’ offering services online but with a dentist and dental clinic. However, there appears to be little difference between this and the other companies as the website states there is no need to visit their practice, at-home impressions are appropriate, but advise they visit their own dentist for a check-up. Any dentist affiliated with them may risk their registration, as lack of face-to-face consultation, informed consent as well as marketing are in conflict with the GDC Standards (BOS, 2020d; GDC, 2013a, 2013b, 2020; The Probe, 2020).

Clinical assessment before any treatment is necessary to address underlying oral health problems, as well as allowing clinician judgement to determine whether proposed orthodontic treatment is safe, suitable and in the patient’s best interests. In their statement on remote treatment, the GDC recommend that ‘for all dental interventions, patients should have a face-to-face consultation with a trained clinician at the beginning of treatment’ (GDC, 2020). This project showed that in many cases DIY-orthodontic providers are offering services that do not include face-to-face contact with an appropriate registrant.

Clinical interaction also gives patients the opportunity to consider the risks and benefits of all available options, provide valid and informed consent, and be satisfied that that a course of treatment proposed is likely to meet their needs and expectations. This project revealed online reviews involving a number of complaints around not achieving the desired results, such as unresolved anterior open bites, ‘gummy smiles’ or complex cases that would realistically be only treated with comprehensive orthodontic and/or orthognathic treatment. There is no opportunity for the DIY providers analysed in this study to assess and discuss such complications, or complex treatment requirements, or discuss what aesthetic result aligning teeth will or will not have on their occlusion or facial profile. This is a significant limitation and would normally be incorporated into the consent process.

The paucity of information concerning the risks of DIY-orthodontics on almost all the websites analysed, representation of benefits only and absence of consent procedures reinforces concerns that consumers are not fully informed of the risks of undertaking orthodontic treatment without supervision. This brings into question whether the informed choice element of the consent process is robust. It is hoped this is discussed to some extent once a consumer engages a provider for aligners. A deficient consent process may mean that many DIY-orthodontic providers may be in breach of the GDC standard 3.1 and 3.2: ‘Obtain valid consent before starting treatment, explaining all the relevant options and the possible costs’ and ‘Make sure that patients understand the decisions they are being asked to make’ (BOS, 2020a, 2020c, 2020d; GDC, 2013a). In the absence of informed choice, valid consent cannot be achieved. This article supports the findings by Meade and Dreyer (2021), which also found the quality of information on DIY-orthodontic websites to be poor, thus meaning consent for aligner treatment based on these websites would be invalid (Meade and Dreyer, 2021)

According to the GDC Standards (2013) and guidance on advertising, all information made public must be truthful and credible (GDC, 2013a, 2013b). Claims made by companies on their websites included that the aligners are ‘0% more comfortable than regular aligners’ (company 7) and they were ‘50% faster than traditional clear braces treatment’ (company 1). On further investigation of the websites, there were no references to any literature supporting this. This is a concern as it could be misleading and without proper explanation or clarification and may influence decision-making. If DIY-orthodontic websites remain unregulated, it leaves open the risk that this advertising does not uphold the GDC standards (GDC, 2013b). It was considered to review these websites’ compliance with the GDC guidance on ethical advertising, as per the study by Parek and Gill (GDC, 2013b; Parekh and Gill, 2014). However, these websites and products are not currently classed as dental services, so this element was discounted; however, this may be a future project if they are reclassified. At present, all websites fail to fulfil these GDC standards, are poor quality and at risk of misleading the public.

Wexler et al. (2020) also found that customers who wished to complain and get a refund from certain companies in the United States had to sign a non-disclosure agreement to stop them from posting negative reviews on social media or websites. With this in mind, it may not be possible to ascertain a true reflection of customer experience on social media platforms, which serve as a main marketing tool for these companies (Wexler et al., 2020).

Remote-orthodontic technology is not without benefit if used safely and appropriately by dentists, to supplement conventional orthodontics, such as with the DentalMonitoring or Smile Tracker apps provided by Align Technology Inc. (Invisalign, 2020). Orthodontists themselves are beginning to utilise this digital technology for the remote monitoring of clear aligners. This technology allows online communication between the patient and orthodontist/dentist. Such advances are advantageous for revolutionising modern digital orthodontic care that is supervised by the orthodontist, especially in rural communities (Invisalign, 2020).

## Conclusion

In total, there were seven websites for DTC aligners available in the UK via search engines on the Internet. The authors predict more will come to the UK if this continues to be an unregulated business, with no accountability for any potential adverse outcomes, especially in the wake of the COVID-19 pandemic where access to dental appointments has been severely affected.

The basic cost estimates of treatment with DIY aligners are in the range of £799–£1599, which make them an attractive alternative to conventional orthodontic treatment with a dental care professional. However, there is a significant trade-off: that there seems to be no-one overseeing the aligner therapy, which gives the opportunity for complications to arise, with either an unsatisfactory outcome or even detriment to oral health.

Information available online raises questions as to whether the members of the public are undertaking a course of orthodontic treatment as patients or are consumers of a product. Wording and information on such websites may need to change if it is the latter, as the limited information available does also cause concern as to whether full informed consent has been taken place.

This project highlights the need for such products and information available on websites to be better regulated in order for the potential consumer to be better able to make an informed decision as to whether to undertake DIY-orthodontics, or seek regulated face-to-face aligner treatment, if these products are to be allowed by the GDC and exist as an option for consumers. They should also undergo the scrutiny of GDC principles for ethical advertising and have high-quality information on their websites to aid informed choices (GDC, 2013b; Meade and Dryer, 2021).
